# Using Semantic Web technologies for the generation of domain-specific templates to support clinical study metadata standards

**DOI:** 10.1186/s13326-016-0053-5

**Published:** 2016-03-03

**Authors:** Guoqian Jiang, Julie Evans, Cory M. Endle, Harold R. Solbrig, Christopher G. Chute

**Affiliations:** Department of Health Sciences Research, Mayo Clinic, 200 First St SW, Rochester, MN 55905 USA; Clinical Data Interchange Standards Consortium (CDISC), Austin, TX USA; Johns Hopkins University, Baltimore, MD USA

**Keywords:** BRIDG, RDF, CIMI, Doman analysis model, Clinical study meta-data standards, Detailed clinical model, Semantic Web technologies

## Abstract

**Background:**

The Biomedical Research Integrated Domain Group (BRIDG) model is a formal domain analysis model for protocol-driven biomedical research, and serves as a semantic foundation for application and message development in the standards developing organizations (SDOs). The increasing sophistication and complexity of the BRIDG model requires new approaches to the management and utilization of the underlying semantics to harmonize domain-specific standards. The objective of this study is to develop and evaluate a Semantic Web-based approach that integrates the BRIDG model with ISO 21090 data types to generate domain-specific templates to support clinical study metadata standards development.

**Methods:**

We developed a template generation and visualization system based on an open source Resource Description Framework (RDF) store backend, a SmartGWT-based web user interface, and a “mind map” based tool for the visualization of generated domain-specific templates. We also developed a RESTful Web Service informed by the Clinical Information Modeling Initiative (CIMI) reference model for access to the generated domain-specific templates.

**Results:**

A preliminary usability study is performed and all reviewers (*n* = 3) had very positive responses for the evaluation questions in terms of the usability and the capability of meeting the system requirements (with the average score of 4.6).

**Conclusions:**

Semantic Web technologies provide a scalable infrastructure and have great potential to enable computable semantic interoperability of models in the intersection of health care and clinical research.

## Introduction

The Biomedical Research Integrated Domain Group (BRIDG) model is a formal domain analysis model for protocol-driven biomedical research, and serves as the semantic foundation for application and message development in the standards developing organizations (SDOs) [1, 2]. The increasing sophistication and complexity of the BRIDG model requires new approaches to the management and utilization of the underlying semantics to harmonize domain-specific standards.

A typical use case for the BRIDG model comes from the Clinical Data Interchange Standards Consortium (CDISC) [3]. CDISC initiated the **S**hared **H**ealth **A**nd Clinical **R**esearch **E**lectronic Library (SHARE) project to build “a global, accessible electronic library, which enables standardized data element definitions and richer metadata to improve biomedical research and its link with healthcare” [4]. In it, CDISC envisioned integrated domain-specific templates built from the classes and attributes from the BRIDG model and ISO 21090 data types as a foundation for the definition of research concepts in the therapeutic target areas.

The CDISC SHARE approach to domain-specific templates has much in common with an international collaboration effort initiated by the Clinical Information Modeling Initiative (CIMI) [5], “an international collaboration that is dedicated to providing a common format for detailed specifications for the representation of health information content so that semantically interoperable information may be created and shared in health records, messages and documents” [6]. While the domain-specific templates defined in CDISC SHARE are focused on clinical research and CIMI is more focused on electronic health records (EHR) and secondary use of EHR data, we see the semantic interoperability of the two models as critical for predictable exchange of meaning between two or more systems in the area of health care and clinical research. We also believe that the emerging Semantic Web technologies based on World Wide Web Consortium (W3C) standards can provide much of the infrastructure and tools needed to accomplish this goal.

The W3C standards include the Resource Description Framework (RDF) and the Web Ontology Language (OWL) [7, 8], which provide a scalable framework for semantic data integration, harmonization and sharing. These technologies are beginning to appear in both clinical research and health care workspaces and have been leveraged in several notable projects, including the UK CancerGrid [9], the US caBIG [10] and the National Center of Biomedical Ontologies (NCBO) [11]. The Semantic Web Health Care and Life Sciences (HCLS) Interest Group has been formed under the auspices of the W3C to develop, advocate for and support the use of the Semantic Web technologies across the domains of health care, life sciences, clinical research and translational medicine [12]. In some of our previous studies, we explored the use of OWL to represent clinical study metadata models such as HL7 Detailed Clinical Models (DCMs) [13] and the ISO/IEC 11179 model [14], and investigated a Semantic Web representation of the Clinical Element Model (CEM) for secondary use of the EHR data [15, 16].

The objective of this study is to develop and evaluate a Semantic Web-based approach that integrates the BRIDG model with ISO 21090 data types to generate domain-specific templates to support clinical study metadata standards development. The main purpose of the tools developed in this study is to support SDOs such as CDISC to create information models that can enable data exchange between clinical care systems (e.g., in a CIMI model) and clinical trial systems (e.g., in a BRIDG model). In it we developed a template generation and visualization system based on an open source Resource Description Framework (RDF) store backend, a SmartGWT-based web user interface, and a “mind map” based tool for the visualization of generated domain-specific templates. We also created a RESTful Web Service informed by the Clinical Information Modeling Initiative (CIMI) reference model for access to the generated domain-specific templates. A preliminary usability study is performed to evaluate the system in terms of the ease of use and the capability for meeting the requirements using a selected use case.

## Background

### BRIDG model

**Fig. 1:**
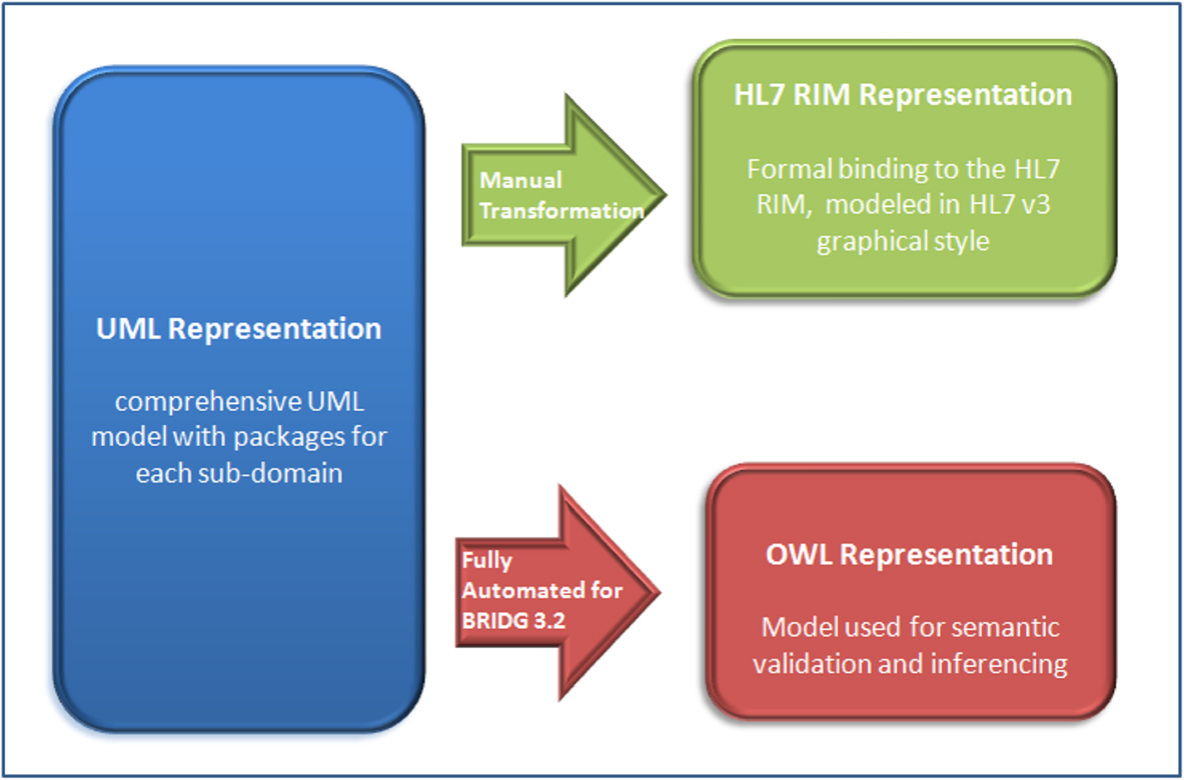
BRIDG multiple-perspective representations in UML, HL7 RIM and OWL

In 2004, CDISC initiated the Biomedical Research Integrated Domain Group (BRIDG) in collaboration with HL7 and National Cancer Institute (NCI). The collaboration effort developed a domain analysis model that is a shared view of the dynamic and static semantics for the domain of protocol-driven research and its associated regulatory artifacts [1]. The BRIDG model was based on the HL7 Development Framework. Multiple representations of the model were introduced in the BRIDG 3.0 release, including the canonical Unified Modeling Language (UML)–based representation, a HL7 Reference Information Model (RIM)-based representation and a ontological representation in a single OWL file. Figure 1 shows BRIDG multiple-perspective representations in UML, HL7 RIM and OWL.

### CDISC standards development

The mission of CDISC is “to develop and support global, platform-independent data standards that enable information system interoperability to improve medical research and related areas of healthcare” [17]. Over the past decade, CDISC has fulfilled its mission by publishing and supporting a suite of standards that enable the electronic interchange of data throughout the lifecycle of a clinical research study [18].

Specifically, CDISC has developed standards for use across the various points in the research study lifecycle:

- Planning: Protocol Representation Model Version 1, which includes Study Design, Eligibility Criteria and Clinical Trial Registration
- Data Collection:
  - oClinical Data Acquisition Standards Harmonization (CDASH) for the collection of data through case report forms
  - oOperational Data Model (ODM) for the collection of operational data through electronic data exchange
  - oLaboratory Model (LAB) for the collection of clinical laboratory data through electronic data exchange
- Data Tabulations
  - oStudy Data Tabulation Model (SDTM) for submission of human subject data to regulatory agencies
  - oStandard for the Exchange of Nonclinical Data (SEND) for submission of non-human subject data to regulatory agencies
- Statistical Analysis: Analysis Data Model (ADaM) for submission of statistical analysis data to regulatory agencies.

### Clinical information modeling initiative

The Clinical Information Modeling Initiatives (CIMI) was officially launched in July, 2011 with more than 23 participating organizations. The initiative was established to “improve the interoperability of healthcare information systems through shared implementable clinical information models” [5]. The principles of the CIMI include “1) CIMI specifications will be freely available to all. 2) CIMI is committed to making these specifications available in a number of formats. 3) CIMI is committed to transparency in its work and product.” The goals of the CIMI include: 1) shared repository of detailed clinical information models; 2) a single formalism; 3) a common set of base data types; 4) formal bindings of the models to standard coded terminologies; and 5) repository is open and models are free for use at no cost. As of May 7, 2013, CIMI is finalizing its reference model specification that consists of a core reference model, a data value type model and a party model.

### Semantic Web technologies

The World Wide Web Consortium (W3C) is the main international standards organization for the World Wide Web [7]. Its goal is to develop interoperable technologies and tools as well as specifications and guidelines to realize the full potential of the Web. The W3C tools and specifications that we used in this study include the Resource Description Framework (RDF) [8], RDF Schema (RDFS) [19], the Web Ontology Language (OWL), OWL 2 [20], the Simple Knowledge Organization System (SKOS) [17], the SPARQL Protocol and RDF Query Language (SPARQL) [21], and the SPARQL Inference Notation (SPIN) [22], which is a W3C Member Submission that can be used to represent SPARQL rules and constraints on Semantic Web models.

## Methods

### System requirements

The system requirements for this study were based on a CDISC SHARE project, in which building domain-specific templates based on BRIDG model is an essential process for clinical study metadata standards development. These requirements include:

- Selection from multiple BRIDG classes. For example, describing a measurement on a subject (such as vital signs like body temperatures) may include the BRIDG classes Defined Observation, Defined Observation Result, Performed Observation, Performed Observation Result and Reference Result.
- Selection of specific attributes from each selected BRIDG class. The attributes include the inherited attributes from its parent classes. For example when selecting attributes based on a BRIDG class Person, the inherited attributes (e.g., *name*, *birthDate*, etc.) from its parent class Biologic Entity shall be available for the selection.
- Specification of the subcomponents of the data type for a specific attribute of a BRIDG class. BRIDG attributes are associated with ISO 21090 data types, each of which has multiple components with its own data type, which may also be a complex. Using the BRIDG class Person as an example, the attribute *educationLevelCode* has the data type *CD. CD*, in turn has a set of components including *code*, *displayName*, *codeSystem*, *codeSystemName*, *codeSystemVersion*, *valueSet*, etc. Each of which components has their own data type.
- Selection of attributes from the BRIDG classes that link to a selected BRIDG class through potential association relationships. For example, through the association “be reported by”, the class Performed Observation links to a set of BRIDG classes including Subject, Healthcare Provider, Laboratory, Device, etc. The attributes from associated classes are available for building a domain-specific template.
- Provide a standard representation of generated templates, which is scalable for supporting downstream development and harmonization of clinical study metadata standards.

### System architecture

**Fig. 2:**
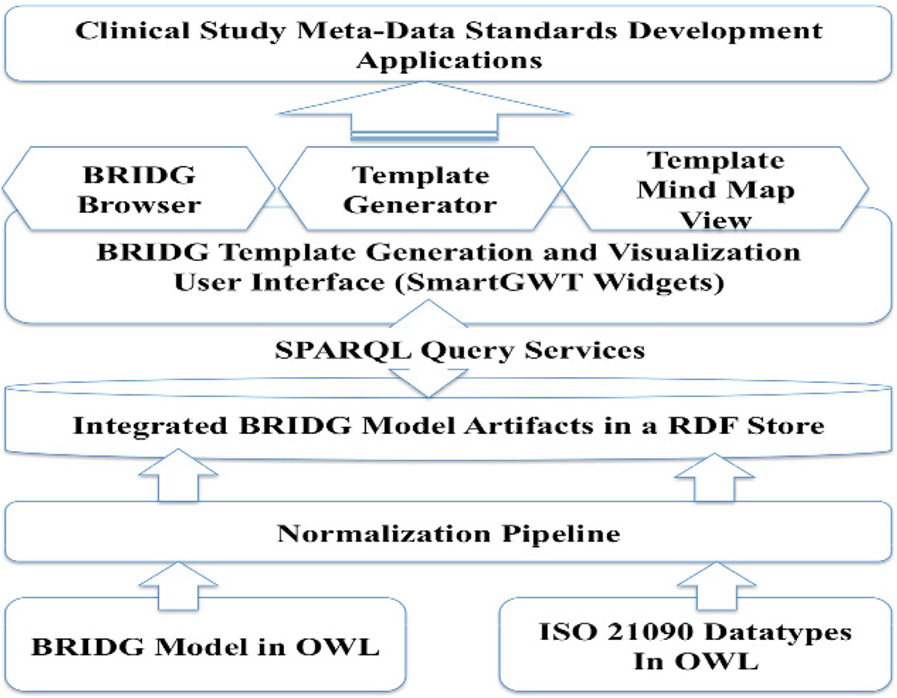
A diagram illustrating the system architecture

Figure 2 shows the system architecture. The system comprises the following modules: 1) a normalization pipeline module; 2) a backend module that uses a RDF store; 3) a frontend module that includes a BRIDG model browser, a template generation mechanism and a mind map viewer for generated templates.

### Implementation

#### Materials

##### BRIDG model in OWL

In the release of the BRIDG version 3.2, an ontological perspective, i.e., OWL representation of BRIDG semantics is developed for the BRIDG model. For this release, the scope of the OWL contents is limited to the information found in the BRIDG UML model. In this study, we used the OWL rendering of the BRIDG model that is publicly available from the release package of the BRIDG 3.2 [1].

##### HL7 V3 data types in OWL

The HL7 OWL project has published an initial draft of the Core HL7 V3 in OWL. The publicly available draft was released on January 2013 and can be downloaded from the HL7 OWL project web site [23]. In this study, we use the HL7 OWL rendering of HL7 V3 data types in place of the ISO 21090 equivalents.

#### Backend implementation

We started with the 4store, an open source RDF store developed at Garlik [24]. We then loaded the RDF image BRIDG model and HL7 V3 data types in OWL into two separate graphs. We also established a SPARQL endpoint that provides standard query services against the RDF store backend.

**Fig. 3:**
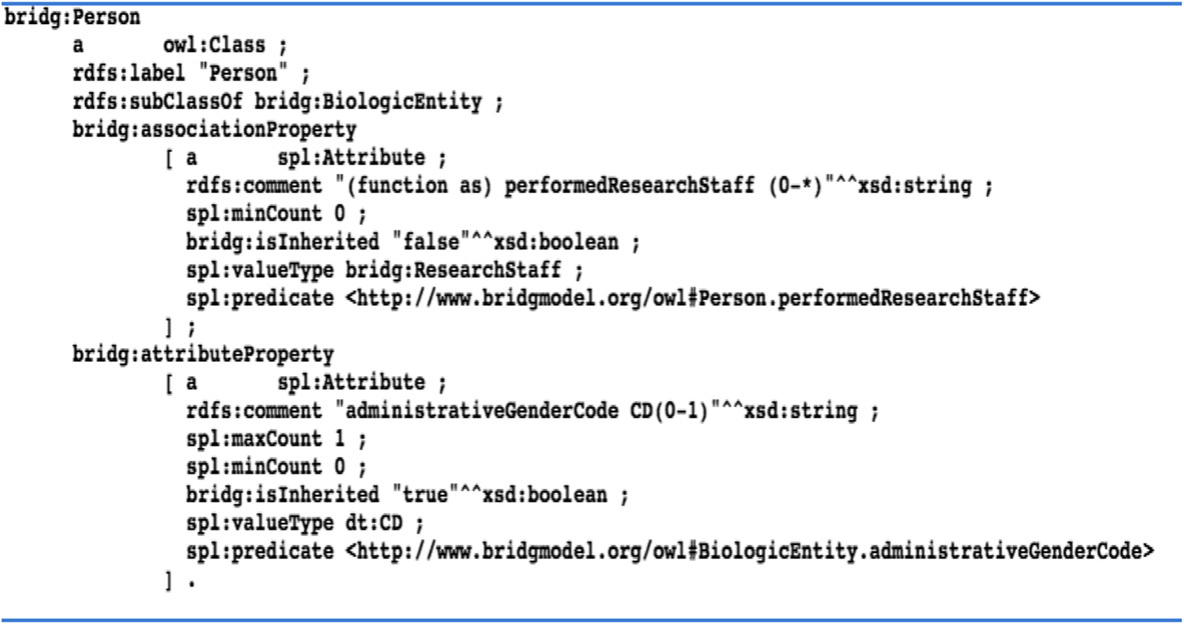
An example of flattened representation for an association and an attribute of the BRIDG class Person using a SPIN template

To make all of the inherited attributes and associations explicit for each BRIDG class, we used Jena ARQ API-based script [25] that recursively retrieved the attributes and associations from parent classes of each BRIDG class and materialized them explicitly using two BRIDG predicates: *bridg*:*attributeProperty* and bridg:associationProperty. We also used a template, *spl*:*Attribute*, from the SPARQL Inference Notation (SPIN) to model the metadata of each attribute and association, including the cardinality and a predicate *bridg*:*isInherited* indicating whether the target attribute or association is inherited or not. Figure 3 shows an example of the flattened representation for an association and an attribute of the BRIDG class *Person*. Following this, we combined the namespaces used for the HL7 V3 data types and the OWL renderings of the BRIDG models.

#### Frontend implementation

##### Building a BRIDG model browser and a template generation mechanism

We developed a BRIDG model browser as a web application based on the SmartGWT API [26]. SmartGWT is a Google Web Toolkit (GWT)-based framework that allows users to utilize its comprehensive widget library for user interface development.

**Fig. 4:**
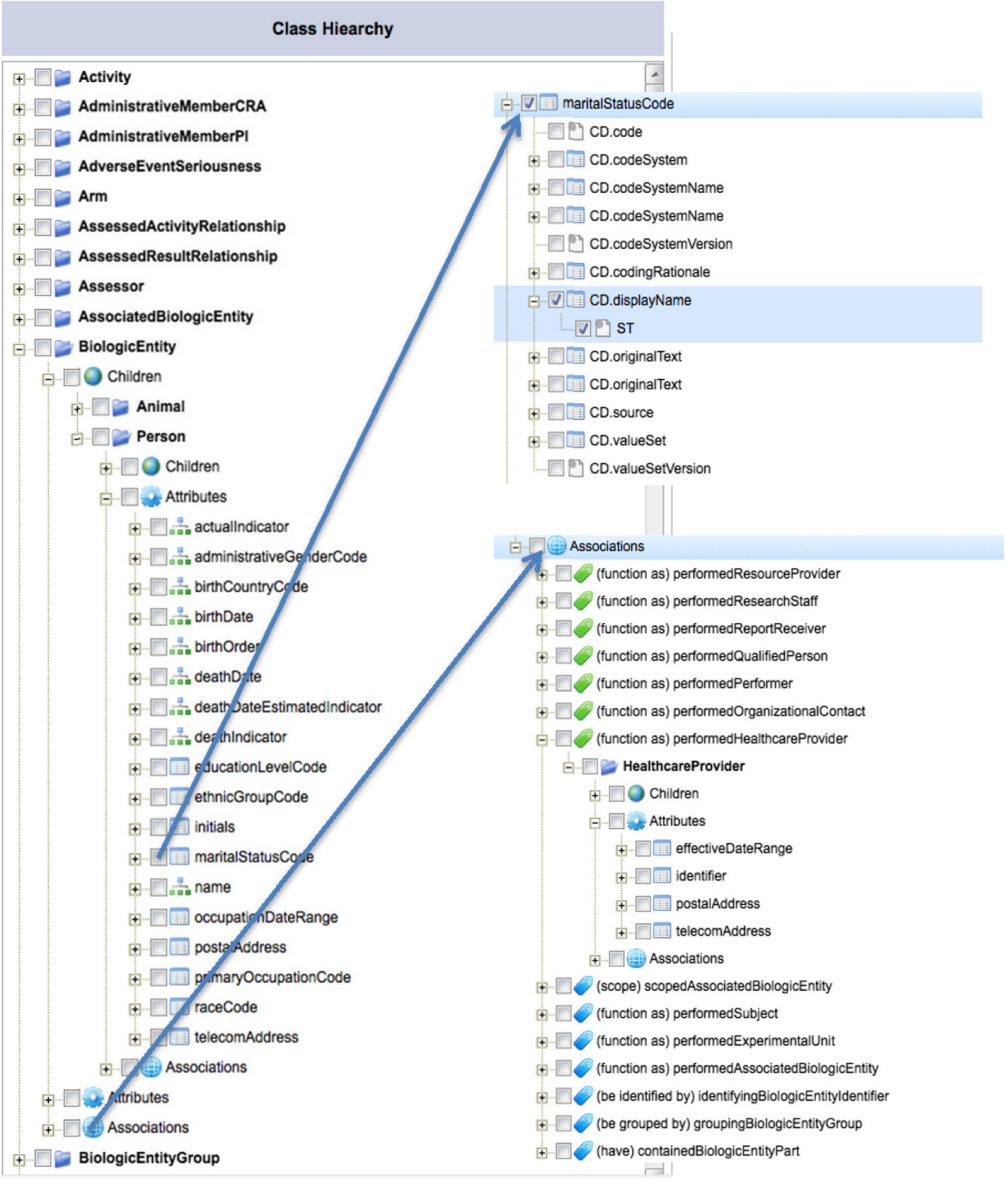
A customized BRIDG model browser with a metadata structure for each class. In the left hand panel, a hierarchical tree of BRIDG classes is displayed. In the right upper part, it displays nested *sub*-*components and their selection for the data type* (*i.e*., *CD*) *of an attribute Person.maritalStatusCode. In the right lower part*, *it displays the associations of the class Person*

**Fig. 5:**
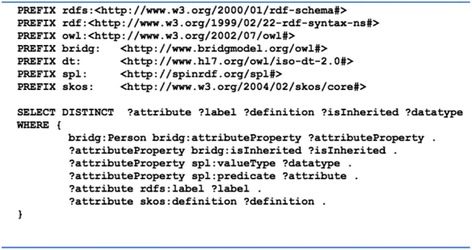
A SPARQL query to retrieve all attributes associated with the BRIDG class *Person*

The browser displays a hierarchical tree of BRIDG classes (see Fig. 4). For each class, the browser displays a metadata structure comprising Children, Attributes and Associations, which streamlined those metadata associated with each class. We defined a set of SPARQL queries to retrieve the children, attributes and associations for each class. Figure 5 shows a SPARQL query to retrieve all attributes associated with the BRIDG class *Person*.

If a BRIDG class has children, they will be displayed under the folder Children. The Attributes folder displays all inherited and non-inherited attributes and their data types. Separate icons are used to differentiate which attributes are local vs. inherited. The sub-components are displayed for complex data types. As an example, the upper right corner of Fig. 4 shows the sub-components of the data type *CD* for the attribute *maritalStatusCode*. Data type sub-components can be expanded to display interior data types.

The Associations folder shows inherited and non-inherited associations with icons representing their inheritance status. The associated class will be displayed and it can be expanded to show its corresponding structure. The lower right hand of Fig. 4 shows the expansion of the Associations folder for the class Person.

We also developed a template generation mechanism by allowing selection of specific elements in the BRIDG model browser. *A target template can be constructed from the attributes* (*including data type components*) *from one or more BRIDG classes*. Based on the system requirements, a set of rules is applied when users make their selections. The upper right hand part of Fig. 4 shows the user selecting the data type *ST* data type of the *CD.displayName* component with the full path of the selected attribute used as the attribute name: *Person.maritalStatusCode.CD.displayName.ST*.

A generated template with a set of selected attributes (including data type components) can be rendered as a “mind map”. We use the Freemind browser [27] to display a target mind map.

##### A CIMI reference model-based Semantic Web representation of generated domain templates

**Fig. 6:**
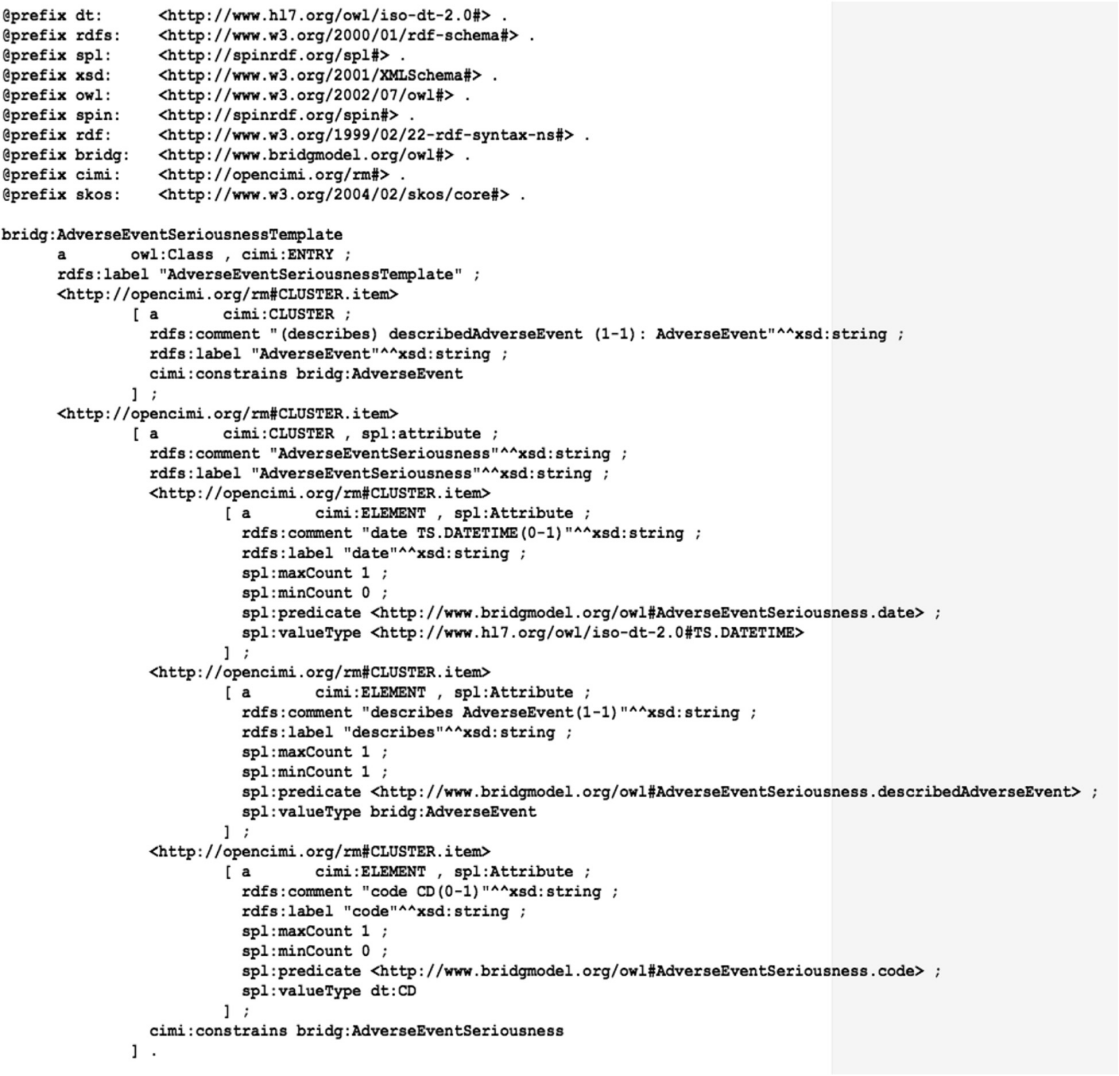
A CIMI-compliant Semantic Web representation in the Turtle format for a domain-specific template generated from the class AdverseEventSeriousness

We created a mapping between CDISC standard objects and CIMI reference model elements. In it a domain-specific template corresponds to the CIMI element *ENTRY* (*the logical root of a single clinical statement within a clinical session*) and the component BRIDG classes and BRIDG attributes correspond to the CIMI element *CLUSTER* (*a set of ELEMENTs*) and *ELEMENT* (*a type of data ITEM*, *which does not itself contain ITEMs*) respectively. Using this mapping, we were able to create a CIMI-complaint Semantic Web representation for generated BRIDG domain-specific templates. Figure 6 shows an example of a CIMI-compliant Semantic Web representation for a domain-specific template generated from the BRIDG class *AdverseEventSeriousness*. As illustrated, we used the elements from the CIMI reference model, such as cimi:*ENTRY*, cimi:*CLUSTER*, cimi:*ELEMENT*, and cimi:CLUSTER.item. We also used the SPIN template *spl*:*attribute* to attach the metadata of each selected attribute including the cardinality.

We then developed the RESTful Web Service that provides programmatic and browser access to the CIMI reference model-based representations of the domain-specific templates. As an example, the CIMI reference model-based representation for the *AdverseEventSeriousness* domain in Turtle format is shown in Fig. 6.

## Results and discussion

### System evaluation

We performed a preliminary evaluation on the system in terms of the usability and the capability of meeting the system requirements as described in the Section 3. For the evaluation design, we created a use case test script that describes the use case of generating a template “Measurement on a Subject”. The target of the use case is to develop a template that covers 5 BRIDG classes, 20 BRIDG attributes and 5 BRIDG associations. We recruited three reviewers: one reviewer (JE, a co-author) from CDISC SHARE team who has extensive expertise on BRIDG model and clinical study metadata standard development, and two other reviewers who are biomedical informatics researchers. We arranged a teleconference meeting and introduced the background of the project and demonstrated the basic features and usages of our frontend widgets to them. We made the web application accessible to the three reviewers who followed the test script to build a template for the target use case. Each reviewer worked individually to complete the test case. After they completed, the three reviewers are asked to answer the evaluation questions in a 1-5 scale, in which 1 stands for “Strongly disagree”, 2 for “disagree”, 3 for “neutral”, 4 for “agree” and 5 for “Strongly agree”. The preliminary results indicated that all three reviewers successfully created the template as described in the test script. All reviewers had very positive responses for the evaluation questions in terms of the usability and the capability of meeting the system requirements (with the average score of 4.6). The reviewers also provided free-text feedback on the system. Some of comments include 1) the suggestion to add a search button for users who look for a particular class and attribute; 2) the suggestion that the icon used for the folder Children could be misleading and confusing; 3) the issues for displaying Freemind map in different browsers; 4) the suggestion of allowing multiple ways to de-select an attribute; 5) the suggestion of allowing to reload the generated template for modification; 6) the suggestion of allowing to constrain the data type of ANY in a specific data type.

### Discussion

In this study, we designed, developed and evaluated a BRIDG-based domain-specific template generation and visualization system for supporting clinical study metadata standards development. We consider that the system and approach developed in this study are significant in both domain specific perspective and technical perspective.

#### Domain specific significance

The system requirements were derived directly from a real-world CDISC SHARE project [4], which demonstrated that a scalable mechanism for access and modular use of the BRIDG model elements is essential for supporting metadata standards development. With the increasing complexity of the BRIDG model, the BRIDG development team has made efforts to deal with the scalability issue. One example is the six subdomain views, Adverse Event, Common, Protocol Representation, Regulatory, Statistical Analysis, and Study Conduct, which help domain experts to navigate subsets of the domain semantics. In addition, multiple representations as described in the Background section are used to meet the requirements from different use cases. In this study, we focused on the domain-specific template generation use case and developed a customized BRIDG browser that enables the standards developer to interact with the BRIDG model elements. Specifically, we streamlined the metadata for each BRIDG class using a metadata structure of Children, Attributes and Associations. The preliminary evaluation demonstrated the positive results in terms of the ease of use and the capability to meet the system requirements. In addition, the generated domain-specific templates can be rendered in a Mind Map view, which has been widely used in the standards development community. Furthermore, we developed a Semantic Web representation informed by CIMI reference model for the generated domain-specific templates, providing a modular representation for a specific domain exposed as a standard RESTful service. This will enable semantic harmonization with other CIMI-compliant models, potentially developed from different contexts.

#### Technical significance

Semantic Web technologies played a critical role in the system design and development. First, the RDF data model and the triple store technology enabled data integration of the BRIDG model and ISO 21090 data type model. All BRIDG attributes have defined data types based on ISO 21090. For those complex data types, they have multiple components. Some of the components of a complex data type are required for a domain-specific template. For example, the *CD* data type has the components *valueSet* and *valueSetVersion* that can be used for the valueset binding. Utilizing the Semantic Web OWL/RDF version of the two models, we were able to seamlessly link the data type defined for each BRIDG attribute with their components defined in the ISO 21090 data type model. Note that we unified the namespaces used for the data types in the two models for the integration purpose.

Second, the subsumption property, *rdfs*:*subClassOf*, asserted in the OWL/RDF version of the BRIDG model provides an elegant way to compute and retrieve the inherited attributes and associations from parent classes for a BRIDG class. The BRIDG model is authored in the UML, in which a child class should inherit all asserted attributes/associations from their parent classes, just as in object-oriented model. Being able to browse and select the inherited attributes/associations is one of key system requirements for domain-specific template generation. As part of the normalization pipeline, we retrieved and materialized all inherited attributes/associations for each BRIDG class, which streamlined the metadata of each BRIDG class and made the attribute selection straightforward to users.

Third, a SPARQL endpoint was established to provide standard SPARQL query services for accessing the content of the BRIDG model elements. We defined a set of SPARQL queries to extract the metadata for each BRIDG class. We found that the normalization pipeline as we implemented it was very helpful to simplify the query building. For example, as we materialized the inherited attributes and associations for each BRIDG class, building the SPARQL queries for retrieving this kind of metadata was simplified. In addition, the SPARQL endpoint based on 4store implementation supports SPARQL 1.1 update features, which enables the storage and update of generated domain-specific templates with their provenance information and provides potential for future authoring application development.

Fourth, a CIMI-compliant Semantic Web representation was developed for representing the generated domain-specific templates and the elements from the CIMI reference model were used. As we mentioned above, the CIMI is finalizing its reference model. A Semantic Web representation of the CIMI reference model and its compliant clinical information models is one of key tasks envisioned by the CIMI community. We consider that our current efforts in this study would provide useful experiences and test cases for the CIMI community. In addition, we used a SPIN template to represent the metadata of an attribute in a domain-specific template. The SPIN framework is designed to represent the SPARQL rules and constraints in Semantic Web models. SPARQL rules are a collection of RDF vocabulary that builds on the W3C SPARQL standard to let us define new functions, stored procedures, constraint checking, and inference rules for Semantic Web models. The rules are all stored using object-oriented conventions and the RDF and SPARQL standards. We expect that the SPIN framework will provide a natural way to represent the constraints and rules in a CIMI-compliant model and enable an automatic mechanism for model validation and consistency checking.

#### Limitations and future study

There are several limitations in the study. First, a more rigorous evaluation from a panel of domain experts from broader communities would be helpful in the future. The system will be iteratively enhanced based on the feedback from the evaluators. For example, the search functionality would be helpful to allow users to find a target class/attribute more quickly. Second, the system evaluation was limited to the ease of use and the fulfillment of those basic requirements. We have not evaluated the system in terms of the CIMI conformance for generated domain-specific templates. We are actively working with the CDISC SHARE and CIMI communities to review the current prototype representation. One of main tasks is to develop the mappings between the ISO 21090 data types used in the BRIDG model and the data type defined in the CIMI reference model.

## Conclusions

In summary, we developed and evaluated a Semantic Web –based approach that integrates the model elements from both BRIDG model and ISO 21090 model and enables a domain-specific template generation mechanism for supporting clinical study metadata standards development. The source code of the application are available from the project GitHub website at https://github.com/caCDE-QA/bridgmodel. We demonstrated that Semantic Web technologies provide a scalable infrastructure and have great potential to enable computable semantic interoperability of models in the intersection of health care and clinical research.

### Availability of supporting data

The data set(s) supporting the results of this article is(are) included within the article (and its additional file(s)).

## Abbreviations

BRIDG: The Biomedical Research Integrated Domain Group
SDO: Standards developing organizations
RDF: Resource Description Framework
CIMI: The Clinical Information Modeling Initiative
CDISC: The Clinical Data Interchange Standards Consortium
SHARE: The Shared Health And Clinical Research Electronic Library
EHR: Electronic health records
W3C: World Wide Web Consortium
OWL: The Web Ontology Language
NCBO: National Center for Biomedical Ontology
HCLS: The Semantic Web Health Care and Life Sciences
DCMs: Detailed Clinical Models
CEM: Clinical Element Model
NCI: National Cancer Institute
UML: Unified Modeling Language
RIM: Reference Information Model
CDASH: Clinical Data Acquisition Standards Harmonization
ODM: Operational Data Model
LAB: Laboratory Model
SDTM: Study Data Tabulation Model
SEND: Standard for the Exchange of Nonclinical Data
ADaM: Analysis Data Model
SKOS: The Simple Knowledge Organization System
SPIN: The SPARQL Inference Notation
GWT: Google Web Toolkit

## References

[1] The Biomedical Research Integrated Domain Group (BRIDG) Model [cited 2012 November 19, 2013]. Available from: http://www.bridgmodel.org/.

[2] Fridsma DB, Evans J, Hastak S, Mead CN (2008). The BRIDG project: a technical report. J Am Med Inform Assoc.

[3] The CDISC [November 6, 2012]. Available from: http://www.cdisc.org/.

[4] The CDISC CSHARE [May 15, 2013]. Available from: http://www.cdisc.org/cdisc-share.

[5] The Clinical Information Modeling Initiative (CIMI) [cited 2012 November 6, 2012]. Available from: http://www.opencimi.org/.

[6] CIMI – initial public statement [cited 2012 November 6, 2012]. Available from: http://omowizard.wordpress.com/2011/12/14/cimi-initial-public-statement/.

[7] Huff SM, Rocha RA, McDonald CJ, De Moor GJ, Fiers T, Bidgood WD (1998). Development of the logical observation identifier names and codes (LOINC) vocabulary. J Am Med Inform Assoc.

[8] Dolin RH, Huff SM, Rocha RA, Spackman KA, Campbell KE (1998). Evaluation of a “lexically assign, logically refine” strategy for semi-automated integration of overlapping terminologies. J Am Med Inform Assoc.

[9] Davies J, CGibbons J, Harris S, Crichton C. The CancerGrid Experience: Metadata-Based Model-Driven Engineering for Clinical Trials. Sci Compt Programming. 2014;89:126–143.

[10] Komatsoulis GA, Warzel DB, Hartel FW, Shanbhag K, Chilukuri R, Fragoso G (2008). caCORE version 3: Implementation of a model driven, service-oriented architecture for semantic interoperability. J Biomed Inform.

[11] Noy NF, Shah NH, Whetzel PL, Dai B, Dorf M, Griffith N, et al. BioPortal: ontologies and integrated data resources at the click of a mouse. Nucleic Acids Res. 2009;37(Web Server issue):W170-3. Epub 2009/06/02. doi: gkp440 [pii] 10.1093/nar/gkp440. PubMed PMID: 19483092; PubMed Central PMCID: PMC2703982. 10.1093/nar/gkp440 PMC2703982 19483092

[12] Nadkarni PM, Brandt CA (2006). The common data elements for cancer research: remarks on functions and structure. Methods Inf Med.

[13] HL7 Detailed Clinical Models [cited 2012 November 6, 2012]. Available from: http://wiki.hl7.org/index.php?title=Detailed_Clinical_Models.

[14] ISO/IEC 11179, Information Technology -- Metadata registries (MDR) [cited 2012 November 6, 2012]. Available from: http://metadata-standards.org/11179/.

[15] Tao C, Jiang G, Oniki TA, Freimuth RR, Pathak J, Zhu Q, et al. A Semantic-Web Oriented Representation of the Clinical Element Model for Secondary Use of Electronic Health Records Data. J Am Med Inform Assoc 2012;(doi:10.1136/amiajnl-2012-001326). 10.1136/amiajnl-2012-001326 PMC3628064 23268487

[16] Tao C, Jiang G, Wei WQ, Solbrig H, Chute CG (2011). Towards Semantic-Web based representation and harmonization of standard metadata models for clinical studies. AMIA Summits Transl Sci Proc.

[17] The Simple Knowledge Organization System (SKOS) [November 6, 2012]. Available from: http://www.w3.org/TR/skos-reference/.

[18] Jiang G, Solbrig HR, Iberson-Hurst D, Kush RD, Chute CG (2010). A collaborative framework for representation and harmonization of clinical study data elements using semantic MediaWiki. AMIA Summits Transl Sci Proc.

[19] The RDF Schema vocabulary (RDFS) [November 6, 2012]. Available from: http://www.w3.org/2000/01/rdf-schema.

[20] The OWL 2 [November 6, 2012]. Available from: http://www.w3.org/TR/owl2-syntax/.

[21] The SPARQL Query Language for RDF [November 6, 2012]. Available from: http://www.w3.org/TR/rdf-sparql-query/.

[22] SPARQL Inference Notation (SPIN) [November 1, 2012]. Available from: http://spinrdf.org/.

[23] HL7 OWL Project [April 10, 2013]. Available from: http://gforge.hl7.org/gf/project/hl7owl/.

[24] 4Store Website [May 8, 2013]. Available from: https://github.com/garlik/4store.

[25] Jena ARQ API [May 15, 2013]. Available from: http://jena.apache.org/documentation/query/.

[26] SmartGWT API [May 15, 2013]. Available from: https://github.com/isomorphic-software/smartgwt.

[27] Freemind [May 15, 2013]. Available from: http://freemind.sourceforge.net/wiki/index.php/Main_Page.

